# Interference of breast implants with echocardiographic image acquisition and interpretation

**DOI:** 10.1186/1476-7120-5-9

**Published:** 2007-02-23

**Authors:** Mohammad-Reza Movahed

**Affiliations:** 1 University of Arizona Sarver Heart Center, Department of Medicine, Section of Cardiology, Tucson, Arizona, USA

## Abstract

Echocardiography is one of the most important diagnostic testing in cardiology. The presence of a breast implant overlying heart can cause significant impairment of the echocardiographic acoustic window. Breast implants are increasing in popularity in the USA and the Federal Drug and Food Administration (FDA) just approved silicone implants again. In this review, the impact of silicone breast implant on the echocardiographic image acquisition and interpretation is discussed.

## Background

Occasionally, I have been called by echocardiographers complaining about technical difficulties they have encountered in women with breast implants and many times I have called echocardiographers and complained about a poor study but later realized that the study was performed in a patient with silicone breast implant with limited window. Breast implants are increasing in popularity [1,2] and FDA recently approved silicone implants for cosmetic use. Echocardiography is one of the most commonly used diagnostic test in cardiology for the evaluation of cardiac structures and has increased dramatically in the last decade. Furthermore, stress echocardiography is commonly used to assess the presence of coronary disease in women with chest pain. Breast tissue is an important cause of false positive stress testing using gated SPECT imaging due to breast attenuation. This limitation is not a significant problem with stress echocardiography which makes stress echocardiography the diagnostic test of choice in women with chest pain. [3] However, the presence of the silicone breast implants can cause major problem in visualizing cardiac chambers and valves and can cause marked impairment of diagnostic window during stress echocardiography for the visualization of wall motions. Despite this commonly observed limitation of acoustic window in patients with breast implants by cardiologists and echocardiographers, there is only one published manuscript available in the literature describing this limitation. [4] With the aging population in the USA, cardiovascular disease is increasing and remains the number one cause of death in women. When today's young women reach older age, limitations in the diagnostic cardiac testing such as echocardiography can contribute to significant increased risk to the patient and cost to society.

### The underlying mechanism of impaired acoustic window caused by breast implant

The underlying physical property of the silicon breast implants that causes interference with the ultrasound beam during echocardiographic examination is not known and has not been studied. Similar to air in the lung but to a lesser degree, silicone breast implants appear to prevent penetration of ultrasound beams. The poor penetration appears to be persistent and unrevealing despite increase in gain or change in the ultrasound wave's frequency. Silicone is not as dense as calcium, and therefore, does not appear to cause significant shadowing that can be seen in calcified structures.

**Figure 1:**
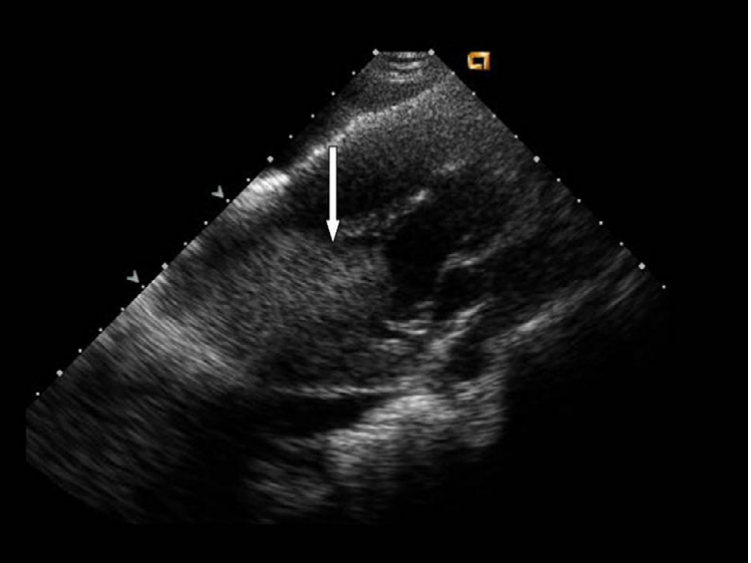
A silicone breast implant in this patient caused marked limitation of echocardiographic acoustic window and image acquisition in the parasternal long axis view obscuring left ventricular cavity and septum.

**Figure 2:**
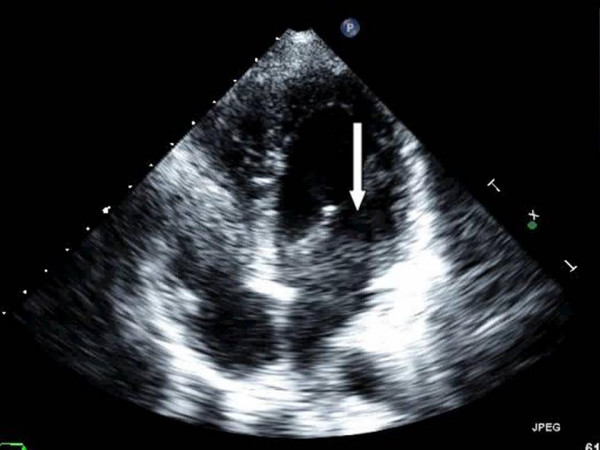
A large shadow is seen across the right and left ventricle caused by the silicone breast implant that limited the echocardiographic window in the 4 chamber view.

**Figure 3:**
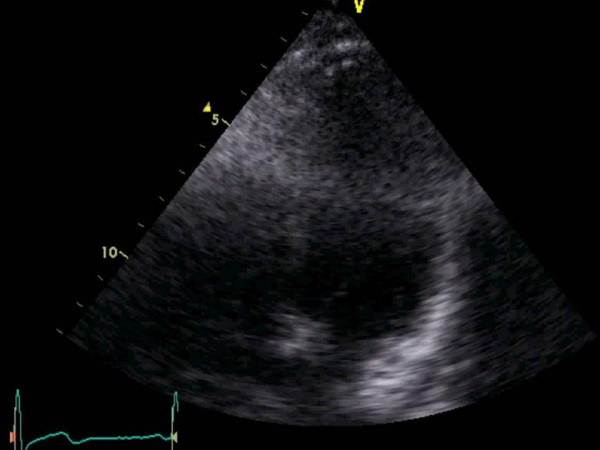
Similar to figure 2, a large shadow is seen across the right and left ventricle secondary to the silicone breast implant in this patient limiting echocardiographic window in the 4 chamber view.

**Figure 4:**
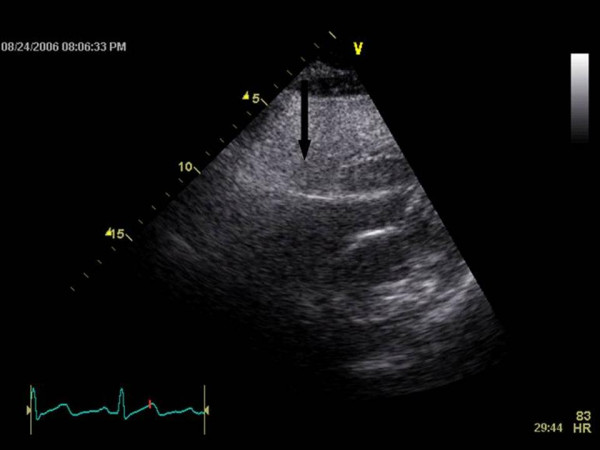
The arrow shows a large shadow and a bright ring obscuring the subcostal acoustic window.

An example of this limitation can be seen in figure 1. Similar to air in the lung, a large shadow obscuring the septal wall and left ventricular cavity can be seen in figure 1. In figures 2 and 3, a silicone breast implant produced a large band-like artifact and limitation of the echocardiographic window that can be seen obscuring right and left ventricular structures in two patients. Even in the subcostal view in figure 4, the echocardiographic window is obscured by a silicone breast implant similar to the lung tissue. None of these artifacts appear to be caused by shadowing that is usually seen in the prosthetic valves or calcified structures. The detailed underlying mechanisms for the poor acoustic window caused by silicone breast implants require future studies.

### What is the solution?

**Figure 5:**
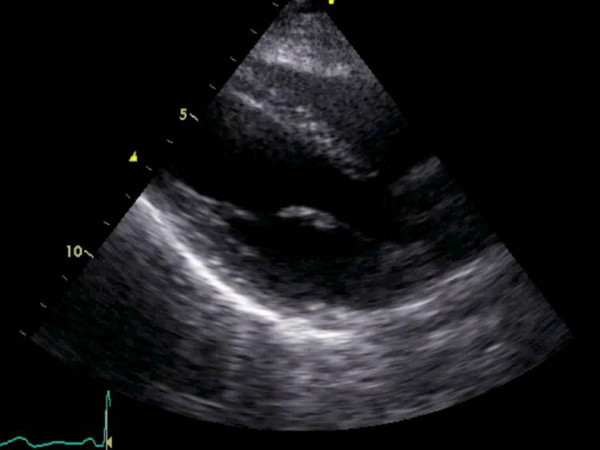
In order to improve the acoustic window, the parasternal long axis view was modified by the echocardiographer causing tilting of the image.

Our understanding about the reason for a poor acoustic window that is caused by silicone is very limited and not known. There is no definite solution to this problem. As the appearance of the artifacts is similar to the artifacts caused by air in the lung, changing the ultrasound beams property should have no significant effect in improving the acoustic window. Changing the position of the heart away from the silicone implant during in- or expiration may improve the acoustic window temporarily. Depending on the size of a breast implant, standard echocardiographic views need to be modified in order to prevent the ultrasound beam crossing the silicon implant. An example of modified parasternal long axis view can be seen in figure 5. Improvement in the acoustic window was achieved at the cost of tilting the image. Subcostal view appears to have the least interference of the ultrasound beam with the breast implant. The four-chamber view needs to be adjusted to more inferior and lateral positioning of the ultrasound probe in order to direct the ultrasound beams beneath the implant. Echocardiographers should notify the interpreting cardiologists about the modifications of the standard views. The position of the probe needs to be described more accurately in order to help the cardiologists in identifying the correct wall segments. Despite repositioning of the probe, commonly echocardiographic images remain suboptimal causing marked limitation in the interpretation of the study.

## Conclusion

Despite the known fact by cardiologists regarding the significant interference of breast implants with cardiac diagnostic testing such as echocardiography during daily work, there is only one study published describing this problem. There is little awareness in the medical community (other than cardiologists) and the population about this fact. The FDA recently approved silicone breast implants for cosmetic reasons without mentioning the diagnostic difficulties that breast implants pose during echocardiographic studies. The medical community and young women considering this cosmetic surgery should be aware and informed about this problem.

## Abbreviations

Federal Drug and Food Administration (FDA)
